# Videooculography “HINTS” in Acute Vestibular Syndrome: A Prospective Study

**DOI:** 10.3389/fneur.2022.920357

**Published:** 2022-07-12

**Authors:** Athanasia Korda, Wilhelm Wimmer, Ewa Zamaro, Franca Wagner, Thomas C. Sauter, Marco D. Caversaccio, Georgios Mantokoudis

**Affiliations:** ^1^Department of Otorhinolaryngology, Head and Neck Surgery, Inselspital, University Hospital Bern and University of Bern, Bern, Switzerland; ^2^Hearing Research Laboratory, ARTORG Center, University of Bern, Bern, Switzerland; ^3^University Institute of Diagnostic and Interventional Neuroradiology, Inselspital, University Hospital Bern and University of Bern, Bern, Switzerland; ^4^Department of Emergency Medicine, Inselspital, University Hospital Bern and University of Bern, Bern, Switzerland

**Keywords:** HINTS, videooculography, acute unilateral vestibulopathy, stroke, vertigo

## Abstract

**Objective:**

A three-step bedside test (“HINTS”: Head Impulse-Nystagmus-Test of Skew), is a well-established way to differentiate peripheral from central causes in patients with acute vestibular syndrome (AVS). Nowadays, the use of videooculography gives physicians the possibility to quantify all eye movements. The goal of this study is to compare the accuracy of VOG “HINTS” (vHINTS) to an expert evaluation.

**Methods:**

We performed a prospective study from July 2015 to April 2020 on all patients presenting at the emergency department with signs of AVS. All the patients underwent clinical HINTS (cHINTS) and vHINTS followed by delayed MRI, which served as a gold standard for stroke confirmation.

**Results:**

We assessed 46 patients with AVS, 35 patients with acute unilateral vestibulopathy, and 11 patients with stroke. The overall accuracy of vHINTS in detecting a central pathology was 94.2% with 100% sensitivity and 88.9% specificity. Experts, however, assessed cHINTS with a lower accuracy of 88.3%, 90.9% sensitivity, and 85.7% specificity. The agreement between clinical and video head impulse tests was good, whereas for nystagmus direction was fair.

**Conclusions:**

vHINTS proved to be very accurate in detecting strokes in patients AVS, with 9% points better sensitivity than the expert. The evaluation of nystagmus direction was the most difficult part of HINTS.

## Introduction

Acute vestibular syndrome (AVS) consists of vertigo, nausea/vomiting, and gait unsteadiness together with head motion intolerance and nystagmus lasting from days to weeks (1). The most common cause of this syndrome is acute unilateral vestibulopathy (AUVP). However, some patients with AVS can suffer from brainstem or cerebellar strokes that mimic AUVP (2). There is a high prevalence of dizziness in the emergency department (ED) (3, 4) with a large proportion of strokes (3)^1^.

The Head-Impulse-Nystagmus-Test-of-Skew (“HINTS”) battery proved to be more accurate in detecting strokes than MRI scan of the brain, especially if it is performed in the beginning of symptoms (5). However, the accuracy of “HINTS” can vary and depends on the experience of the physician (6). Moreover, although “HINTS,” since its first description is thought to have been established in the clinical practice and a bedside three-step examination seems to be a very fast and easy way to detect a stroke, physicians in the ED are still not so familiar with this examination (7–9). In addition, the sensitivity and specificity of head impulse varies with experience, and even experts have difficulties (10) and need a learning curve (11).

Nowadays, the use of VOG devices assists physicians to quantify eye movements. These devices are easy to use (11) and they can serve in the near future with telemedicine and machine learning (12) for remote areas or in pandemic times (13, 14) as a diagnostic tool for acute dizziness and as a support for physicians in the ED analog to an “Eye-ECG”(15). Although there are many studies that show the superiority of video head impulse test (vHIT), there are no studies that assess the aggregated results of all the other steps of the HINTS battery using VOG.

In this study, we sought to assess and compare the diagnostic accuracy of VOG “HINTS” (vHINTS) and of clinical “HINTS”(cHINTS) in predicting a stroke in the ED. Furthermore, we wanted to calculate the concordance between clinical and VOG-assisted tests for each of the three steps of the “HINTS” examination.

## Materials and Methods

### Patient Characteristics

In this prospective, cross-sectional study, data from patients with AVS (convenience sample) were collected in the ED between July 2015 and April 2020 and were part of a larger study (DETECT: Dizziness Evaluation Tool for Emergent Clinical Triage) (16–18). The inclusion and exclusion criteria have been described previously (18). The accuracy of vHIT, video test of skew, and nystagmus test for discriminating vestibular strokes as a single stand-alone test, has been evaluated and previously published (16, 19). Here, we present the data from patients with AVS who received all the three tests clinically (cHINTS) and with VOG (vHINTS) at the bedside. A neurootologist (expert) with 2 years of experience in the field, performed the physical examination with cHINTS assessment and vHINTS testing in all the enrolled patients. We performed caloric testing in all the patients as an additional examination at the time of ED presentation either in the ED or in our vertigo center. All the patients received an acute MRI either within 48 h in the ED or a second, delayed MRI (3–10 days after onset of symptoms) if there was no acute MRI indicated based on clinical grounds or if the first acute MRI was non-diagnostic with regard to the question of a stroke. The delayed MRI served as a gold standard for stroke detection. Patients with a negative acute and/or delayed MRI and a pathological caloric test were diagnosed as having acute unilateral vestibulopathy (AUVP)/vestibular neuritis. Additionally, we collected information on age and gender. All the enrolled patients gave written consent. The local ethics committee (IRB) approved this study (KEK # 047/14).

### MR Protocol

The patients were scanned at one of our six MR scanners either on a 1.5 T scanner (Siemens MAGNETOM Avanto and Siemens MAGNETOM Aera; Siemens Medical Solutions, Erlangen, Germany) or a 3 T scanner (Siemens MAGNETOM; Siemens Medical Solutions, Erlangen, Germany). Our standard MRI protocol for all the patients included axial diffusion-weighted imaging (DWI) with apparent diffusion coefficient (ADC) [5 mm slice thickness (SL)], axial fluid-attenuated inversion recovery (FLAIR) (5 mm SL), axial susceptibility-weighted imaging (SWI) (1.6 mm slice thickness), and time of flight (TOF) angiography (0.5 mm slice thickness). Optional and depending on the clinical symptoms, axial brainstem diffusion-weighted imaging (DWI) with apparent diffusion coefficient (ADC) (3 mm SL) and axial T2-weighted imaging over the brainstem (3 mm SL) were added. After the application of intravenous gadobutrol (Gadovist; Bayer Schering Pharma, Berlin, Germany) in an antecubital vein with a 5-ml/s injection rate, we acquired a standard dynamic susceptibility contrast (DSC) MRI perfusion (5 mm slice thickness) as well as a contrast-enhanced T1 turbo spin echo (TSE)-weighted sequence (slice thickness 5 mm SL). Finally, contrast-enhanced magnetic resonance angiography (CE MRA) of the head and neck vessels was acquired after injection of a second bolus of gadobutrol with a 3-ml/s injection rate. If indicated, follow-up MR imaging was performed using the same MR scanner and field strength with the same MRI protocol or a short native variant of the MR protocol without acquisition of sequences with contrast.

### vHINTS

**Figure 1:**
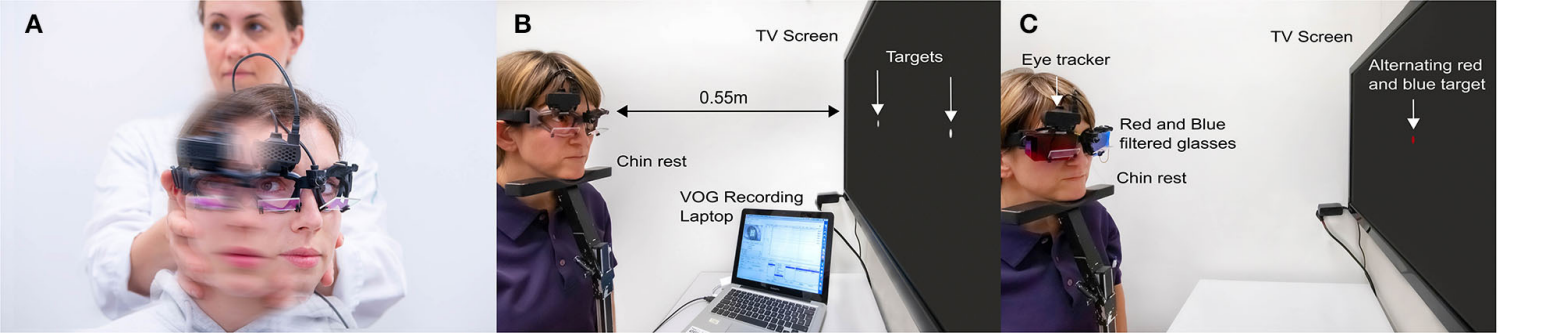
Illustration of all parts of the Head Impulse-Nystagmus-Test of Skew (HINTS) battery by videooculography (VOG). **(A)** Video head impulse test, **(B)** videonystagmography, and **(C)** video test of skew.

We recorded vHINTS with a VOG device (EyeSeeCam, Munich) and measured head and eye movement velocity (head impulse test), nystagmus slow phase velocity (SPV), and vertical ocular misalignment (test of skew) with a head-mounted infrared high-speed camera (monocular, 250 Hz) connected to a laptop by USB (Figures 1A–C). The high-speed infrared camera was calibrated by projecting dots on a TV screen or a tablet with a predefined distance.

vHIT was performed by fast passive horizontal head movements (high frequency, 10–20° head excursion in 100–300 m/s corresponding to 1,000–6,000°/s^2^ acceleration) in room light during visual target fixation at more than 1 m distance. VOR gain values were derived from eye velocity divided by head velocity at 60 ms after HIT onset.

For nystagmus quantification (beating direction and SPV), we used three fixation lights as a target for straight-ahead gaze and for eccentric gaze positions of 15 ± 5 deg to the right and left. We recorded an average SPV for 10 s in each target position (tablet: distance eyes to target: 260 mm, target size: 4 mm, luminosity: 6.17 Lux, angular size: 0.89 degrees; TV screen: distance eyes to target: 55 cm, target Size: 5 mm, luminosity: 11.8 Lux., angular size: 0.23 degrees).

Vertical ocular misalignment was tested by fixating an alternating colored dot (red/blue) displayed for 2 s in the center of the TV screen or the tablet while synchronizing with VOG. We used color-filtered glasses on both eyes (red filter for the left eye and blue filter for the right eye), allowing only a monochromatic view of the target dot. Skew deviation was quantitatively reported in degrees (eye position) or converted into prism diopters. Details about automated skew deviation calculation have been described elsewhere (20). Here, we report skew deviation in degrees (vertical misalignment) throughout the manuscript.

### Statistical Analysis

All statistics were reported using the SPSS statistical software (IBM SPSS Statistics for Windows, Version 25.0. Armonk, NY, IBM Corp.). We classified the patients into central or peripheral “HINTS” (binary outcome) based on vHINTS or cHINTS exams. We used VOG cut-off values for vHINTS classification derived from previous studies: head impulses with bilateral vHIT VOR gain larger than >0.685 (17) and/or skew deviation larger than 3.3 deg (16) and/or any change in nystagmus beating direction (19) were classified as central. We conducted cross-tabulations to assess the specificity (Spec) and sensitivity (Sens) for tests such vHIT alone, combination of vHIT and videonystagmography (vHINT), or all the three tests including video test of skew (vHINTS). Accuracy, Sens. and Spec. were also calculated for cHIT (clinical HIT), cHINT (clinical HIT and nystagmus test), and cHINTS. Cohen's Kappa was calculated for the assessment of agreement between expert's evaluation and VOG.

## Results

### vHINTS vs. cHINTS

**Figure 2:**
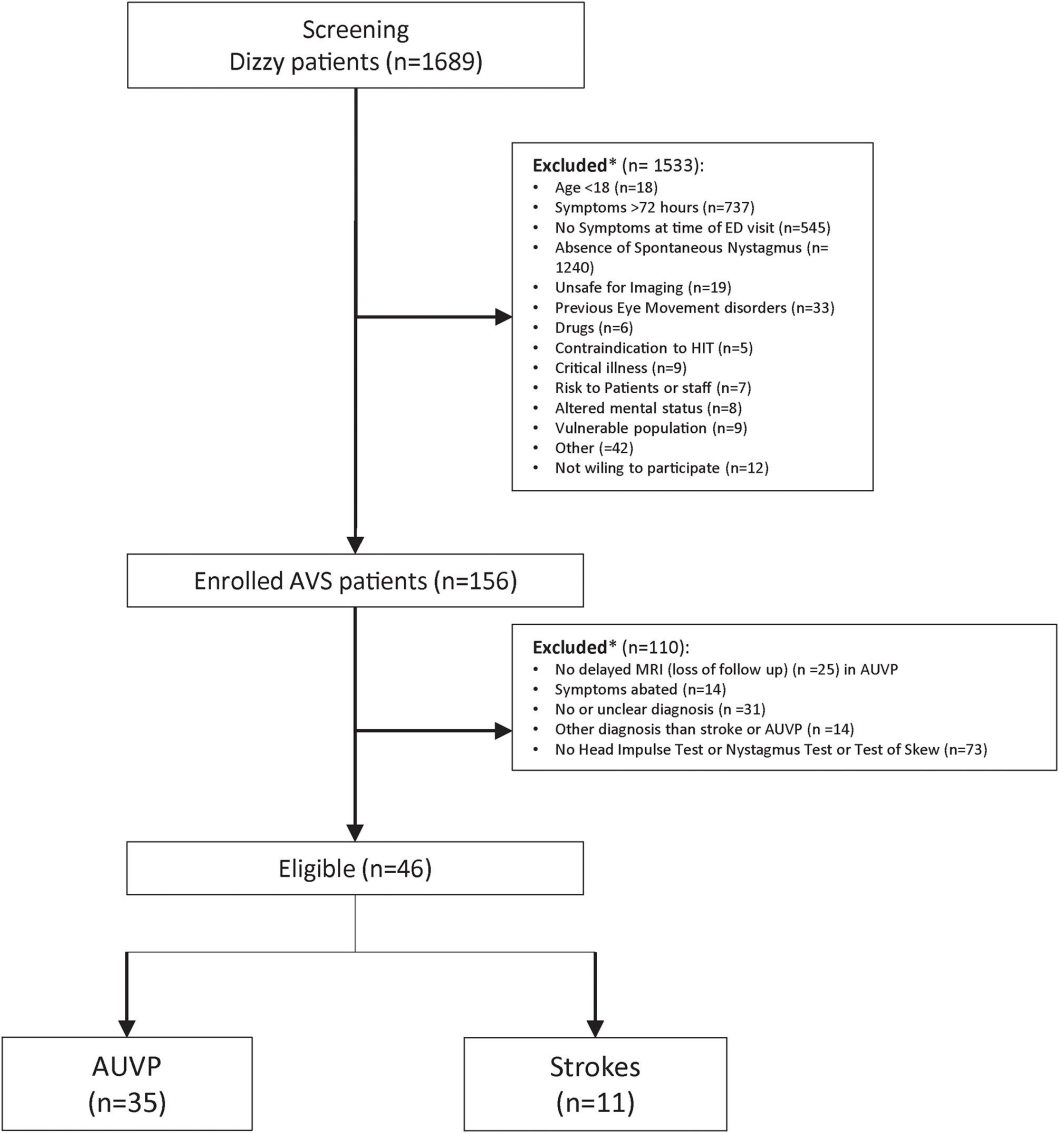
Flowchart of all screened patients with dizziness and the inclusion and exclusion processes. *Patient could have had one or several reasons for exclusion.

We analyzed the data from 46 patients (21 women and 25 men aged between 30 and 78, mean 55 ±15 y) with a diagnosis of stroke or AUVP and who completed cHINTS and vHINTS measurements (35 with AUVP and 11 with stroke) (Figure 2). Details of patient diagnosis, vascular territories of strokes, and findings of the clinical tests are shown in Supplementary Table S1.

**Figure 3:**
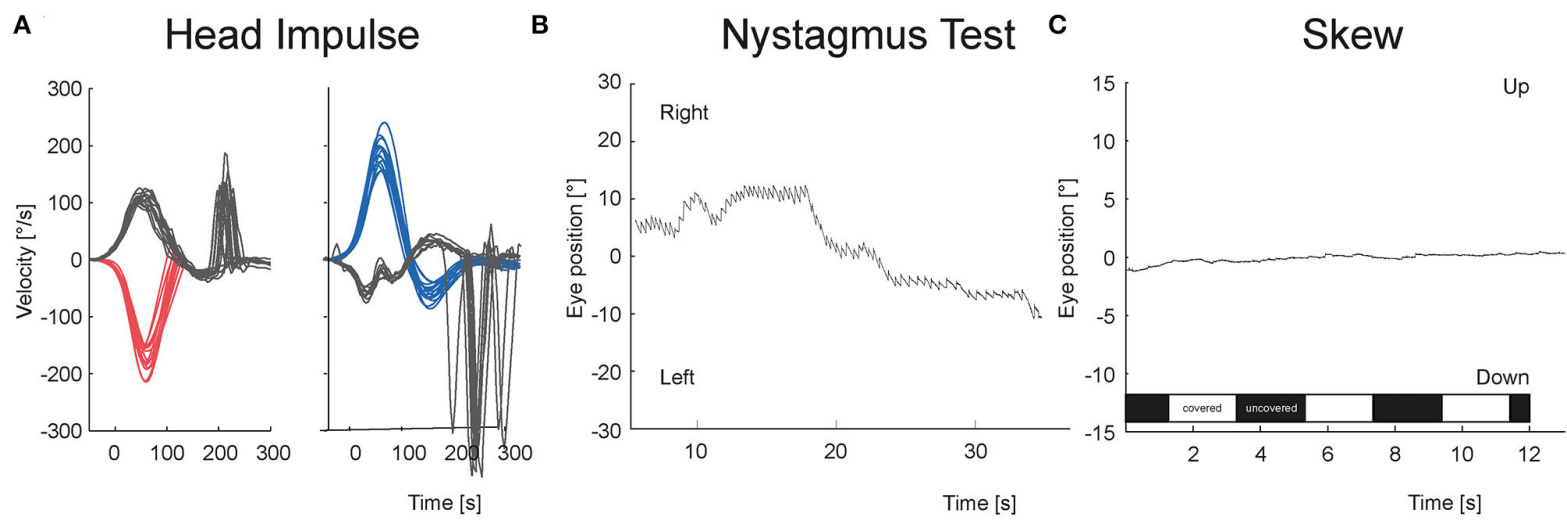
VOG eye recordings of a patient with AUVP are shown. **(A)** vHIT is abnormal to the left with corrective saccades. **(B)** Nystagmus is unidirectional at all gaze directions. Panel **(C)** There is no vertical eye movement in test of skew regardless of whether the right eye was covered (white bar) or uncovered (black bar).

**Figure 4:**
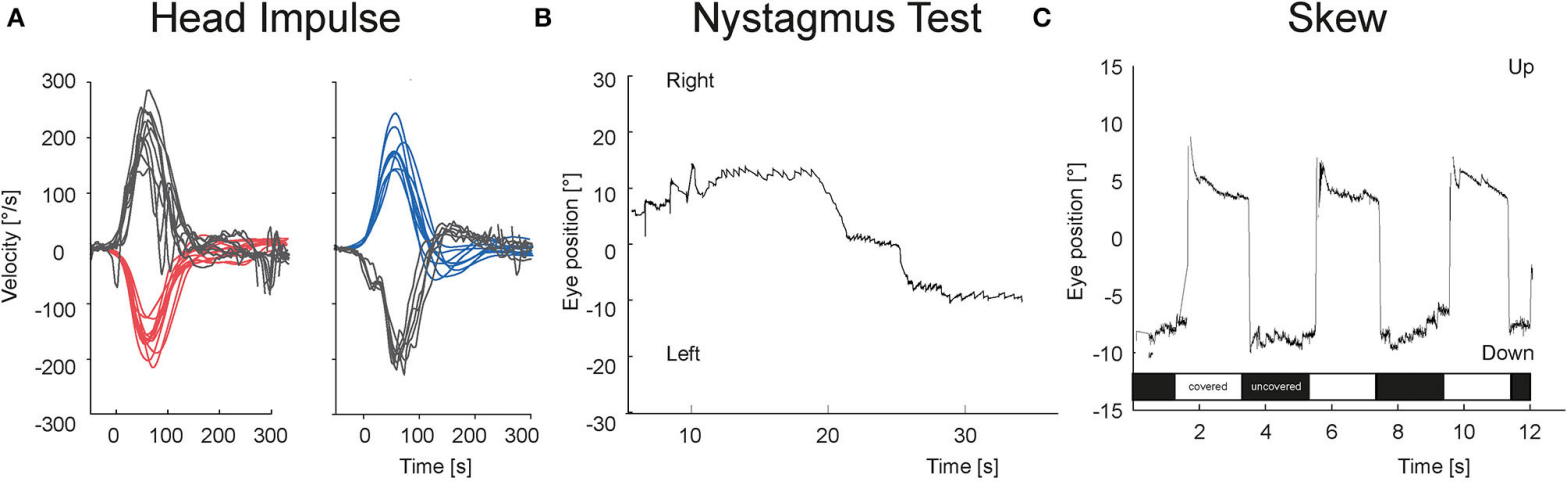
VOG eye recordings of a patient with stroke. **(A)** vHIT is bilaterally normal without any corrective saccades. **(B)** Nystagmus is changing the beating direction at right and left gaze. **(C)** There is a vertical eye movement of 11 degrees in the test of skew during the transition from covered (white bar) to uncovered (black bar) right eye and vice versa.

Figures 3, 4 show the VOG eye recordings of a patient with AUVP and a patient with stroke. Typically, a patient with AUVP has an abnormal head impulse test with asymmetrical VOR gain and corrective saccades, unidirectional nystagmus, and no skew deviation (Figure 3). On the other side, a patient with stroke can appear with a normal VOR gain bilaterally without corrective saccades or direction changing nystagmus or skew deviation (Figure 4). Any of these signs are red flags for stroke.

**Figure 5:**
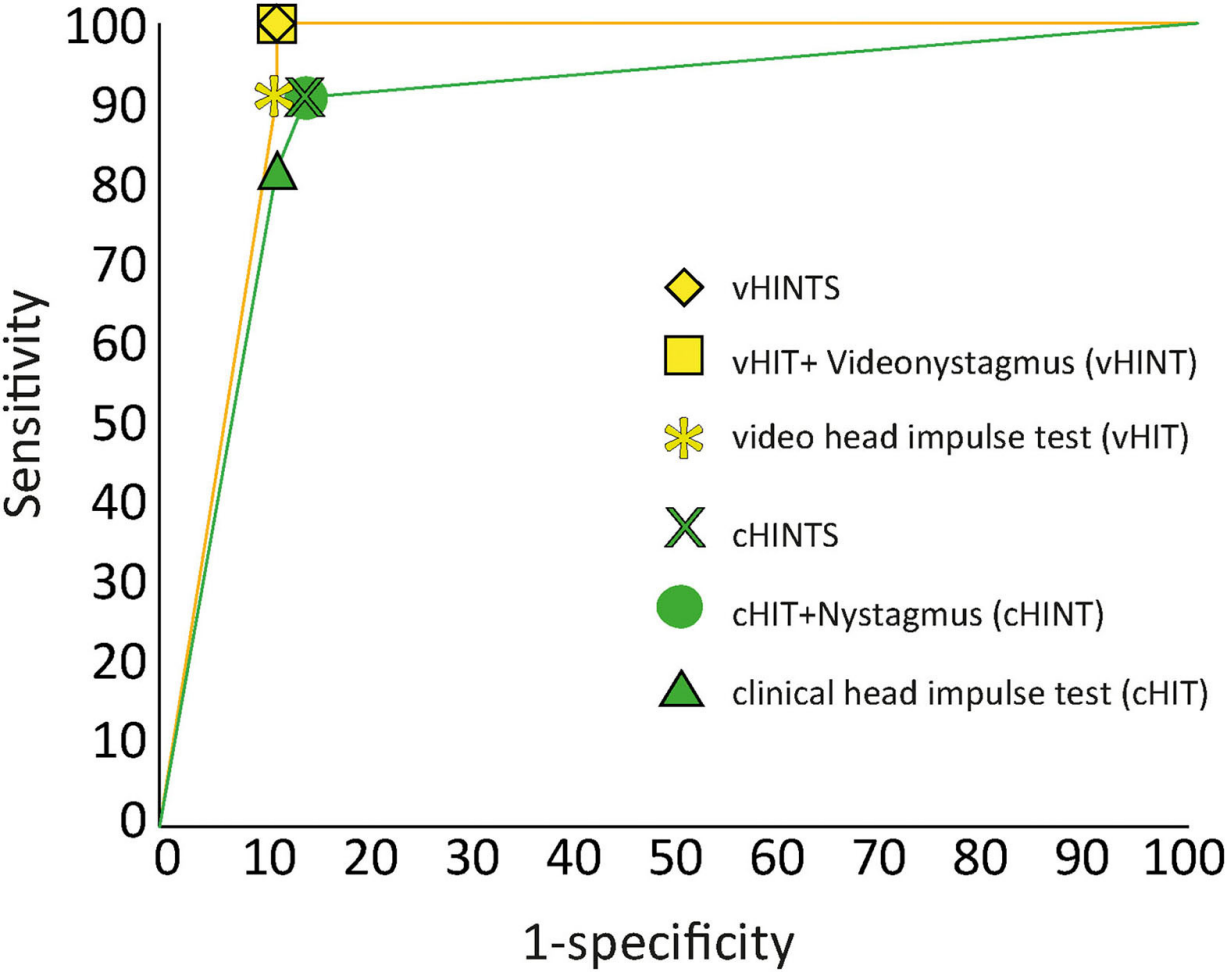
ROC analysis for stroke diagnosis of clinical (green) vs. video “HINTS” (yellow). We report here the sensitivity/specificity of the head impulse tests alone (HIT) or in conjunction with the other two components of the “HINTS” battery (HINT or HINTS).

Figure 5 shows the receiver operator characteristic (ROC) curve for the overall “HINTS” (clinically and video-assisted) sensitivity and specificity for stroke in patients with AVS. It also shows the sensitivity of the HIT alone (vHIT sensitivity was 91% and specificity 89%) or in conjunction with the assessment of nystagmus beating direction at eccentric gaze (vHINT/cHINT). Overall, cHINTS sensitivity was 90.9% and specificity was 85.7%, whereas vHINTS sensitivity was 100% and specificity was 88.9%. False positive rate was 14.3%, and false negative rate was 9.1 % for clinical evaluation. False positive rate was 11.1%, and false negative rate was 0% for VOG. Accuracy was 88.3% for clinical evaluation and 94.2% for the VOG.

There was a perfect agreement between clinical test of skew and video test of skew (*k* = 1) and a good agreement for head impulse test (cHIT vs. vHIT) (*k* = 0.63). Physicians had more difficulties in detecting direction-changing nystagmus, which was clearly detectable with videonystagmography resulting in a fair agreement (*k* = 0.284).

## Discussion

Our study demonstrates that vHINTS has a perfect sensitivity in predicting posterior circulation strokes and proved to be even better than the expert. On the other side, VOG is not the gold standard test to recognize AUVP, and almost 11% of peripheral cases can be misclassified as strokes. Evaluation of direction-changing nystagmus was the most challenging of the three HINTS steps.

Video HIT alone has a 91% sensitivity, which is, according to the literature, even better than an early DWI MRI (5). The specificity of vHIT is 89%, and it does not change if we add nystagmus or test of skew, as there is a case of AUVP with normal vHIT but hypofunction in caloric test. Dissociation between abnormal calorics and normal vHIT can also be seen in patients with mild vestibular hypofunction. Thus, patients presenting with a clinical picture of AVS, with a normal delayed MRI (3–10 days after symptom onset) and a normal vHIT, would be good candidates for further investigations by calorics (17).

On the other hand, cHIT showed a good agreement with vHIT. Its execution and test result evaluation remains challenging even for experts (10). There are many reasons for this. First and foremost, it is not always easy to perform large head acceleration on patients with acute dizziness. What is more, spontaneous nystagmus and covert saccades make things more complicated. With regard to HIT, the use of VOG is mandatory.

However, our study results showed that detection and interpretation of nystagmus continue to pose challenges. Discernment of nystagmus seems to be difficult because of low-intensity nystagmus in patients with stroke (18), which is sometimes evaluated as physiologic gaze-evoked nystagmus (GEN). VOG distinguishes physiologic GEN from pathologic GEN by calculation of time constant. Time constant is defined as the reciprocal value of the increase in SPV (drift) per increase in degree of gaze eccentricity, and reflects the fidelity of the neural gaze-holding integrator (19). In addition, VOG can quantify more accurately the nystagmus suppression test, which is an additional useful test for stroke detection (18). Frenzel glasses are much less sensitive than VOG (21).

Furthermore, we showed that the test of skew in the HINTS test is the simplest step to perform clinically in the ED, since there was a perfect agreement between clinical test of skew and video test of skew. This is not surprising, because only large skews are considered as a red flag for stroke and are discernable/visible to the examiner without any VOG support (16).

## Strengths and Limitations

The examiner who performed the VOG measurements is an expert in the field. It is still unclear whether or not the results can be generalized for non-experts. As we have shown in our previous studies, although the detection of a saccade is challenging even for very experienced examiners (10), the performance of a vHIT seems to be easy after a brief instruction by an expert (11).

We used a non-commercial VOG system with projected and synchronized gaze targets on a screen that are not available in current VOG systems in the market. There is clear superiority in detecting strokes using VOG; however, current available systems do not offer an automated quantitative analysis of all three HINTS steps, and there is no automated interpretation of test results.

Another limitation of our study is the high proportion of exclusions (Figure 2). Many patients without a clear diagnosis and with missing or invalid VOG results were excluded. This may lead to potential selection bias; thus, we should be prudent when generalizing the results. However, technical issues and invalid recordings happened randomly in unselected patients.

Since 2009, when Newman-Toker first recommended the use of VOG devices as an ECG analog for the eyes, many studies have proved the accuracy and feasibility of using these devices (9, 16, 17, 19, 22, 23).

Non-experts might benefit even more from vHIT (9), since it offers a standard examination less dependent of the examiners' experience; however, it remains operator-dependent because eye-tracking systems are susceptible to artifacts (24, 25). Non-experts struggle with the use of such systems and its interpretation. Here, telemedicine can solve the problems as long as there are no automated systems in the market (26, 27). This may overcome the lack of expertise outside metropolitan areas. Furthermore, intensive educational courses for ED physicians through vertigo experts are an option. Application of artificial intelligence on big patient's data in the future can lead to development of an accurate automated interpretation of VOG results (12, 24, 28).

## Implications for Clinicians

Our findings have practical implications for clinical care. Clinical HINTS may not always be diagnostic for vestibular stroke in patients with AVS in the ED because of its lower sensitivity than vHINTS. We therefore strongly recommend the use of a VOG device for all the three parts of the “HINTS” protocol. vHINTS could be a potent and cost-efficient diagnostic tool for smaller community hospitals without 24-h MRI service with no experts available, in rural hospitals, in underserved areas, or in resource-poor nations.

ED physicians should become familiar with the application and interpretation of vHINTS in order to minimize diagnostic errors. We also recommend the implementation of a dizziness telemedicine service to support ED physicians in the diagnostic process.

## Conclusions

vHINTS had a high accuracy in detecting central causes of AVS. Its accuracy exceeds that of expert's clinical examination. Nystagmus evaluation was the most difficult part of the three-step test without the use of the VOG device. VOG devices should be used in the future in EDs.

## Data Availability Statement

The original contributions presented in the study are included in the article/Supplementary Material, further inquiries can be directed to the corresponding author/s.

## Ethics Statement

The studies involving human participants were reviewed and approved by KEK Bern, #047/14. The patients/participants provided their written informed consent to participate in this study. Written informed consent was obtained from the individual(s) for the publication of any potentially identifiable images or data included in this article.

## Author Contributions

AK and EZ collected and processed the data. GM and AK conceived the study, analyzed, interpreted the data, and wrote the draft. MC, TS, WW, and FW were involved in the interpretation of the data and in the review. All authors discussed the results, commented on the manuscript, read, and approved the final version.

## Funding

This study was supported by the Swiss National Science Foundation (#320030_173081).

## Conflict of Interest

The authors declare that the research was conducted in the absence of any commercial or financial relationships that could be construed as a potential conflict of interest.

## Publisher's Note

All claims expressed in this article are solely those of the authors and do not necessarily represent those of their affiliated organizations, or those of the publisher, the editors and the reviewers. Any product that may be evaluated in this article, or claim that may be made by its manufacturer, is not guaranteed or endorsed by the publisher.

## Footnotes

^1^Nikles F, Kerkeni H, Zamaro E, Korda A, Sauter TC, Kalla R, et al. Do stroke patients with dizziness present a vestibular syndrome without nystagmus? An underestimated entity (2022).

## Abbreviations

AVS: acute vestibular syndrome
AUVP: acute unilateral vestibulopathy
“HINTS”: Head-Impulse-Nystagmus-Test-of-Skew
ED: emergency department
VOG, videooculography, vHIT: video head impulse test
cHINTS: clinical HINTS
vHINTS: VOG “HINTS”.
