# Supplementary material for: Videooculography “HINTS” in Acute Vestibular Syndrome: A Prospective Study
Source: Front Neurol. 2022 Jul 12;13:920357. doi: 10.3389/fneur.2022.920357 (PMC9314570; doi:10.3389/fneur.2022.920357)
Supplement: Supplementary file 1 [file Data_Sheet_1.PDF]

Supplementum

Table S1. Diagnosis and clinical test results

Patient Number	Diagnosis	Video Head Impulse Test	Video Gaze Holding Nystagmus	Video Test Of Skew	Clinical Head Impulse Test	Clinical Gase Holding Nystagmus	Clinical Test Of Skew	Vascular Territory Of Stroke
1	AUVP	0*	0	0	0	0	0	
2	AUVP	0	0	0	0	0	0	
3	AUVP	0	0	0	0	0	0	
4	AUVP	0	0	0	0	0	0	
5	AUVP	0	0	0	0	0	0	
6	AUVP	0	0	0	0	0	0	
7	AUVP	0	0	0	0	0	0	
8	AUVP	0	0	0	0	0	0	
9	AUVP	0	0	0	0	0	0	
10	STROKE	1	1	0	1	1	0	PICA/SCA/VA/BA/PCA**
11	AUVP	0	0	0	0	0	0	
12	AUVP	1	0	0	1	0	0	
13	STROKE	1	0	0	1	0	0	SCA
14	AUVP	0	0	0	1	0	0	
15	AUVP	1	0	0	1	0	0	
16	STROKE	1	0	0	1	1	0	PICA
17	AUVP	0	0	0	0	1	0	
18	STROKE	1	1	0	1	0	0	BA
19	STROKE	1	0	0	1	1	0	BA
20	AUVP	0	0	0	0	0	0	
21	AUVP	0	0	0	0	0	0	
22	AUVP	0	0	0	0	0	0	
23	AUVP	0	0	0	0	0	0	
24	AUVP	0	0	0	0	0	0	
25	AUVP	0	0	0	0	0	0	
26	AUVP	0	0	0	0	0	0	
27	STROKE	1	0	0	1	1	0	PICA
28	STROKE	0	1	0	1	0	0	PICA
29	STROKE	1	0	0	1	1	0	PICA
30	AUVP	0	0	0	0	0	0	
31	STROKE	1	0	0	1	0	0	PICA
32	AUVP	0	0	0	0	0	0	
33	AUVP	0	0	0	0	0	0	
34	AUVP	1	0	0	0	0	0	
35	AUVP	0	0	0	1	0	0	
36	AUVP	0	0	0	0	0	0	
37	AUVP	0	0	0	0	0	0	
38	AUVP	0	0	0	0	0	0	
39	AUVP	0	0	0	0	0	0	
40	AUVP	0	0	0	0	0	0	
41	AUVP	0	0	0	0	0	0	
42	AUVP	0	0	0	0	0	0	
43	STROKE	1	0	0	0	0	0	PICA
44	AUVP	0	0	0	0	0	0	
45	STROKE	1	1	1	0	1	1	AICA
46	AUVP	1	0	0	0	0	0	

*0: peripheral 1: central
**PICA: posterior inferior cerebellar artery, AICA: anterior inferior cerebellar artery, SCA: superior cerebellar artery VA: vertebral artery BA: basilar artery
